# The Opium Problem

**Published:** 1894-02-03

**Authors:** 


### The Hospital

An Institutional Journal of

The Medical Sciences and Hospital Administration, with

AN EXTRA NURSING SUPPLEMENT.

Vol. XV.—No. 384.] [Feb. 3, 1894.

### The Opium Problem.

M. Renan thought the world was made for men of science. Sectaries, especially if they happen to be missionaries, think it is made to be finally absorbed by their particular ecclesiastical organisation. It is thus that extremes meet. The eager religionists who desire to convert India and China by force of arms, that is, by Act of Parliament, will do well to illuminate themselves for a brief period at the blazing sky-rocket of Renan's colossal and ridiculous egotism. According to M. Challemel-Lacour, it was Renan's conviction that "Science is the task, and will remain the privilege, of an infinitely small number of minds, to whom the rest of the world must be sacrificed. The masses—that is to say, almost the entirety of mankind—are the soil necessary for the growth and prosperity of a handful of thinkers." It would be superfluous to set over against this magnificent dictum any one particular utterance of religious sectaries. All the world knows, however, that the feeling of the true sectary, whether he be Mohammedan or Christian, Catholic or Protestant, is, that all mankind were made to be finally brought to the pronouncement of one particular "shibboleth," on pain of eternal oblivion, or something worse.

In asking our religious fellow-countrymen to try to look upon the opium question, as it affects India, from the standpoint of the physical health and happiness of the natives of India themselves, as well as from the standpoint of the sectarian's interpretation of a noble religion, we present M. Renan for their preliminary consideration. If Renan's sublime egotism produces upon their minds the same kind of effect as it has produced upon the writer's, they will approach the consideration of the question in a temper the exact opposite of that which runs away with the confident dogmatist. There are two primary ideas which, it seems to us, ought never to be overlooked in seeking a righteous solution of problems of this order. The first is, that if there be a Deity, every single human being in the universe must be, and must always have been, equally the object of His regard and care; and the second, that when a body of men profess that their sole *raison d'être* is the welfare and happiness of others, they must insist upon putting themselves, at any rate in imagination, in the place of those others, in order that they may clearly understand the conditions which would make for their welfare and happiness.

The natives of India have been given into the hands of England in the course of human evolution, at any rate for a period. What shall we do with them? "Make them virtuous," says the religionist; "make them healthy and happy," says the physiologist. Both surely are right! But two or three hundred millions of people cannot be made either healthy or virtuous by a mere stroke of the pen. The problem of the health and virtue of the races which inhabit India is gigantic, and can only be solved by minds which at least try to be gigantic too. The religion which the missionaries of this country carry to India assumes that care of the body is reasonable and right. Modern science has discovered, moreover, that unhealthiness is a fruitful source of irreligion. If then this country is to educate India into the highest religion known to the races of men, that is undoubtedly the Christian, it will do well to conserve for the peoples of India all those customs which are necessary to their physical well-being and to their wholesome mental solace.

At the present moment, as most of our readers know, there is sitting in India a Royal Commission whose business it is to investigate the whole question of opium in its relations to producers, consumers, and the revenues of the Indian Government. It is imperative, therefore, that Parliament and the electors of this country should look at the question from every standpoint, because in all probability the issue of the Commission's report will be the signal for Parliamentary action of some kind. The country has looked at the question from the point of view of the religious propagandist—a very proper point of view—for many years. But now let it also patiently consider the problem from the standpoint of the educated and reasonable European doctor. Let it be remembered that the European doctor cannot possibly have any "axe to grind" in this matter. He is not an opium grower, he is not an opium seller, nor is he an opium consumer. He is simply a man whose duties place him in the most intimate of all kinds of contact with the actual life, habits, health, and moral condition of all classes of the natives of India. For real knowledge of the actual facts of the inner life of India, who is there among Europeans to be compared with European doctors who practise in India? We submit then that the doctor's judgment in this matter is entitled to the most serious consideration at the hands both of Parliament and those who make Parliament.

What, in a sentence, are the main facts of the opium question as it affects India and the duty of England? The main facts are that opium is used very extensively throughout India as a stimulant, just as we use alcohol; that used in moderation it is a harmless stimulant; that in many instances it is a beneficial stimulant; but that in many instances also it is not used but abused, and so becomes injurious and dangerous. If these propositions be carefully studied, it will be seen that everything which has been said of opium can be said with equal propriety of alcohol. But does any statesmanlike person propose to suppress the manufacture of beer, wine, or spirits, or to subject the consumers of these articles to pains and penalties? In comparing the use of opium as a stimulant in India with the use of alcohol as a stimulant in England, it is both interesting and important to consider the extent to which the consumption of their respective national stimulants prevails in the two countries. Many people will be surprised when they are told the real facts on this point. Opium is sold and consumed in public shops in India as alcohol is sold and consumed in public-houses in England. Surgeon-General Mouatt, a well-known authority, tells us that whilst there are 184,743 drink shops in Great Britain, there are only 10,417 licensed opium shops in the whole of British India. But as India has a population six times greater than that of this country, it is manifest that the licensed drink shops in this country, in proportion to population, are over a hundred times more numerous than the licensed opium shops in India. In other words, where every native of British India has one shop at which he can purchase his national stimulant, Great Britain has a hundred for the sale of hers. The conclusion to be drawn from this is that the consumption of opium in India is not carried out on a scale which is anything like extensive; that, in fact, if Britain, as a whole, is a moderate and fairly sober nation, British India, as a whole, is at least a hundred times more sober: or to put it in another way, where India has one drunkard, whose ruin is caused by opium, Great Britain has a hundred whose ruin may be caused by drink.

The opium question in India is, after all, a practical one; and in deciding what is to be done, we repeat that statesmen and electors should give due weight to the experienced and entirely disinterested testimony of the European doctors who practise or have practised in India. When men like Sir George Birdwood and Sir William Moore lead the way, and are followed by a practically unanimous profession, in declaring that opium when properly used is not only harmless but in many cases beneficial, and that its abuse is comparatively rare, it is surely incumbent upon practical persons to do nothing to check its legitimate use. If it be asked, What is to be done to check its abuse? the answer is that the abuse must be checked by moral means, exactly as we try to diminish drunkenness, or any other evil of its class in our own country, by instruction, persuasion, and other moral methods. The conversion of India to Western ideas and ways is a mighty, perhaps an impossible, task. But if it is ever to be accomplished, it will be by education and gradual development, not by legal enactment and coercion. In the meantime, the more excellent way with the native Indian races is to put ourselves in imagination in their places, to respect all their harmless beliefs and practices, to treat them as friends and not as slaves, and to raise them gradually to the nobler level of Christian faith and practice by the Christian method of doing unto them exactly as we should wish to be done by if we were compelled in actual reality to change places with them.

